# Supplementary material for: Content-rich biological network constructed by mining PubMed abstracts
Source: BMC Bioinformatics. 2004 Oct 8;5:147. doi: 10.1186/1471-2105-5-147 (PMC528731; doi:10.1186/1471-2105-5-147)
Supplement: Additional File 5 — The original Chilibot query results of the term "long-term potentiation (LTP)" and 22 other terms, limiting the latest references analyzed to the years 1990, 1995, 2000, and 2004. [file 1471-2105-5-147-S5.bz2 › chilibotAdditionalFile5/ltp2000/html/ERK_ACTIN.html]

 


 **ERK** and **ACTIN** 
  
Found 83 abstracts in PubMed,  **30 abstracts were retrieved and analyzed**.  


---

 Search Google  |
 PDF files only 
|  EDU domain only 

---

**Interactive relationship** (e.g. stimulation, inhibition, etc)

- The MEK 1 inhibitor PD98059 decreased  **erk**  1 and 2 phosphorylation and blocked  **actin**  reorganization in a dose dependent manner.  Ref: 11006227 Invest Ophthalmol Vis Sci, 2000
- Our results, therefore, suggest that disruption of  **actin**  cytoskeleton induces chondrogenesis of mesenchymal cells by activating PKCalpha and by inhibiting  **Erk**  1 signaling.  Ref: 10873653 Biochem Biophys Res Commun, 2000
- The localization of smooth muscle alpha  **actin** , myosin heavy chain isoform SM2, and vimentin in explant isolated rat SMCs was affected by a substrate of fibronectin and laminin and also by  **ERK**  MAP kinase inhibitor PD098059 and P38 MAPK inhibitor SB203580.  Ref: 11001858 Exp Mol Pathol, 2000
- During cell migration,  **ERK**  and CAS Crk coupling operate as distinct signaling pathways that facilitate  **actin**  myosin motor assembly and  **actin**  membrane ruffles, respectively.  Ref: 10852105 Immunol Res, 2000
- PI 3 kinase and  **erk**  1 and 2 signaling pathways are activated and necessary for collagen binding and integrin mediated  **actin**  reorganization in embryonic avian corneal epithelium.  Ref: 11006227 Invest Ophthalmol Vis Sci, 2000
- Putative upstream events in the activation of focal adhesion proteins including  **actin**  cytoskeletal reorganization and myosin light chain MLC phosphorylation were also not prevented by inhibition of  **ERK**  activation.  Ref: 10737896 J Cell Physiol, 2000
- ErbB2 facilitates cell invasion through extracellular regulated kinase  **ERK**  activation and coupling of the adaptor proteins, p130CAS and c CrkII, which regulate the  **actin**  myosin cytoskeleton of migratory cells.  Ref: 10648571 J Cell Biol, 2000
- We show that disruption of the  **actin**  cytoskeleton by cytochalasin D causes a complete inhibition of FAK but does not inhibit Shc signaling and activation of  **ERK** .  Ref: 10976102 J Biol Chem, 2000
- A population of  **ERK**  enriched cells was identified as alpha  **actin**  positive smooth muscle cells SMCs.  Ref: 10634796 Arterioscler Thromb Vasc Biol, 2000
- **Erk**  and PI 3 kinase are necessary for collagen binding and  **actin**  reorganization in corneal epithelia.  Ref: 11006227 Invest Ophthalmol Vis Sci, 2000
- Caldesmon is a substrate for extracellular signal related kinases  **ERK**  and p38 MAP kinases in vitro and has been suggested to modulate  **actin**  myosin interaction and contraction.  Ref: 10843887 Am J Physiol Heart Circ Physiol, 2000
- These data indicate that the integrity of  **actin**  filament arrays and the recruitment of IRAK into focal adhesions are involved in the restriction of IL 1 signaling to  **ERK** .  Ref: 10823834 J Biol Chem, 2000

**Parallel relationship** (e.g. studied together, co-existance, homology, etc.)

- In the present study, we examined possible involvement of protein kinase C PKC and extracellular signal regulated protein kinase  **Erk**  1 in chondrogenesis of mesenchymal cells induced by disruption of  **actin**  cytoskeleton.  Ref: 10873653 Biochem Biophys Res Commun, 2000
- We, therefore, investigated the organization of  **actin** , FAK, paxillin, and activated  **ERK**  and activated p38 during Caco 2 motility across collagen I, fibronectin, laminin, and tissue culture treated glass.  Ref: 11054869 Microsc Res Tech, 2000
- Cells treated with latrunculin B or swinholide A, which caused a progressive depolymerization of  **actin**  filaments, showed a reduction or elimination of IL 1 induced  **ERK**  activation, respectively.  Ref: 10823834 J Biol Chem, 2000
- Cryocut sections were stained using antibodies directed against c Jun, phosphorylated c Jun, c Fos, c Jun amino terminal kinase JNK, extracellular signal related kinase  **ERK** , von Willebrand factor, ki67 antigen, and alpha  **actin** .  Ref: 10619996 Eur J Clin Invest, 2000
- FCS evoked both  **ERK**  and p70S6K activity, peaking at 20 40min, and simultaneously increased cardiac myocyte protein synthesis evaluated by 3H leucine incorporation and total cellular protein content, cell size evaluated by morphometry and fluorescence activated cell sorter analysis and expression of a fetal isoform of the muscle specific gene skeletal alpha  **actin**  SKA.  Ref: 10981855 Jpn Circ J, 2000
- The tissues were evaluated for collagen stimulated  **actin**  reorganization,  **erk**  1 and 2 and PI 3 kinase activity, total filamentous  **actin**  accumulation, and collagen binding.  Ref: 11006227 Invest Ophthalmol Vis Sci, 2000
- Expression of  **actin** , FAK, paxillin, phospho  **ERK** , and phospho p38 were examined by immunofluorescent staining in static and motile cells.  Ref: 11054869 Microsc Res Tech, 2000
